# Volatile Organic Compounds from Orchids: From Synthesis and Function to Gene Regulation

**DOI:** 10.3390/ijms21031160

**Published:** 2020-02-10

**Authors:** Mummadireddy Ramya, Seonghoe Jang, Hye-Ryun An, Su-Young Lee, Pil-Man Park, Pue Hee Park

**Affiliations:** 1 Floriculture Research Division, National Institute of Horticultural and Herbal Science, RDA, Wanju-gun, Jellabuk-do 55365, Korea; ramya87.4u@gmail.com (M.R.); hryun@korea.kr (H.-R.A.); lsy8542224@korea.kr (S.-Y.L.); pmpark@korea.kr (P.-M.P.); 2 World Vegetable Center Korea Office (WKO), Wanju-gun, Jellabuk-do 55365, Korea; seonghoe.jang@worldveg.org; 3 Department of Horticultural Science and Biotechnology, Seoul National University (SNU), Seoul 08826, Korea

**Keywords:** *Cymbidium*, floral scents, Orchidaceae, pollination, volatile organic compounds

## Abstract

Orchids are one of the most significant plants that have ecologically adapted to every habitat on earth. Orchids show a high level of variation in their floral morphologies, which makes them popular as ornamental plants in the global market. Floral scent and color are key traits for many floricultural crops. Volatile organic compounds (VOCs) play vital roles in pollinator attraction, defense, and interaction with the environment. Recent progress in omics technology has led to the isolation of genes encoding candidate enzymes responsible for the biosynthesis and regulatory circuits of plant VOCs. Uncovering the biosynthetic pathways and regulatory mechanisms underlying the production of floral scents is necessary not only for a better understanding of the function of relevant genes but also for the generation of new cultivars with desirable traits through molecular breeding approaches. However, little is known about the pathways responsible for floral scents in orchids because of their long life cycle as well as the complex and large genome; only partial terpenoid pathways have been reported in orchids. Here, we review the biosynthesis and regulation of floral volatile compounds in orchids. In particular, we focused on the genes responsible for volatile compounds in various tissues and developmental stages in *Cymbidium* orchids. We also described the emission of orchid floral volatiles and their function in pollination ecology. Taken together, this review will provide a broad scope for the study of orchid floral scents.

## 1. Introduction

The Orchidaceae family is one of the largest and widely diverse families of flowering plants, with more than 28,000 accepted species spanning 763 genera [1]. These plants are absent only in polar and desert regions, but are particularly abundant in the wet tropics worldwide [2]. However, a majority of orchids are distributed locally and generally rare [3]. Associated with the massive number of species in Orchidaceae, orchids display extraordinary floral diversification and represent a highly advanced and terminal line of floral evolution in the monocotyledons. As fascinating and highly popular plants, orchids are valued because of their exquisite flowers and long floral lifespan. These plants consist of great diversity in floral form, size, color, fragrance, and texture. A specific interaction between a pollinator and orchid flower may be one of the factors that promote orchid species richness [4]. The Orchidaceae family can be categorized into four subfamilies (Cypripedioideae, Epidendroideae, Orchidoideae, and Vanilloideae) [5] and comprises a considerable diversity in life forms, with approximately 30% of species being terrestrial and mainly growing as epiphytes and lithophytes [6]. Furthermore, commercial production of orchids has greatly expanded and become a very profitable industry. Dominant species, such as those of *Cymbidium*, *Paphiopedilum*, and *Phalaenopsis*, are cultivated based on consumer flower preferences [7].

Orchids have complex life histories and diversified adaptation strategies; consequently, researchers have focused on orchid flower development and orchid pollination interactions. Flower color and scent are main traits for many floricultural crops. Floral scents emit various types of volatile organic compounds (VOCs). Orchids currently account for a prominent share of the world’s flower trade, with annual sales of more than $4 billion (USD). It is widely used in perfumes, cosmetics, florivores, and medicinal applications. Some are also used as food and traditional medicines in many countries [8]. For example, dried vanilla seed pods (especially *Vanilla planifolia*) are commercially important as a flavoring used in baking, as well as for perfume manufacturing and aromatherapy [9]. *Gastrodia elata* is one of three orchids listed in the earliest known Chinese Materia Medica, and is used for treating headaches, dizziness, tetanus, and epilepsy [10]. However, because of its economic value in floral and pharmaceutical industries, *G. elata* has suffered great losses in habitat, resulting in a rare species [11,12].

Flower color and volatile compounds are key characteristics for many floricultural crops. Synthesis of VOCs occur in all plant organs, including roots, stems, leaves, seeds, fruits, as well as flowers, which are reported to emit the highest amounts and diversity of VOCs [13,14]. To date, more than 1700 floral VOCs have been identified in around 1000 seed plants [14]. In general, VOCs formed in other organs, apart from flowers, are involved in defense mechanisms. Although floral volatiles play a crucial role in reproductive process by attracting pollinators, they also have other adaptive roles [15,16], such as repellents [17,18,19] and physiological protectors against abiotic stresses [14,20]. In addition, floral volatiles are widely used as components of perfumes, cosmetics, flavorings, and even for therapeutic applications. Together with floral color, volatiles emitted by flowers represent key floral signals used by insects to detect and select rewarding flower species [21,22]. Floral scents emit different types of VOCs. VOCs are generally lipophilic and have low molecular weights and high melting points. Based on their origin, function, and biosynthesis, floral scents are grouped into three major clusters: terpenoids, phenylpropanoids, and fatty acid derivatives. Floral volatiles with terpene synthases (TPSs) have been identified in orchids [23,24].

Various species with large genomes are observed in monocots, such as species in Alliaceae, Asparagaceae, Liliaceae, Melanthiaceae, and Orchidaceae [25]. Among these, Orchidaceae, with genome sizes in a 168-fold range (1C = 0.33–55.4 pg), are perhaps the most diverse angiosperm families [25]. Epidendroideae, in Orchidaceae, contain variable genome sizes, with genome sizes in a range of over 60-fold (1C = 0.3–19.8 pg). Orchidoideae, with the largest descending/offspringing from species in subtribe Orchidinae, are pictured by a more restricted range of genomes (1C = 2.9–16.4 pg). Cypripedioideae show genome sizes in only a 10-fold range (1C = 4.1–43.1 pg). Cypripedioideae contain the largest mean genome size (1C = 25.8 pg) among all the subfamilies. Some species in Vanilloideae have been estimated, ranging from 1C = 7.3 to 55.4 pg. *Pogonia ophioglossoides* presents the largest genome size (1C = 55.4 pg) in this family [25]. Apostasioideae, the primitive subfamilies, contain calculated 1C-values ranging from 0.38 to 5.96 pg in a close to 16-fold range [26].

Orchids are one of the most diversified angiosperms and have mesmerized botanists for centuries. For orchids, floral color, shape, and fragrance are primary key determinants of consumer preferences. Many floricultural crops have lost their scents, following traditional breeding. However, only a few genomic resources are available for these non-model plants. Despite its economic as well as biological importance, metabolic engineering approaches on floral scents are still at the stage of infancy in orchids. In this review, we give an overview of orchid floral volatiles with a focus on *Cymbidium* orchids; we review their importance in pollination ecology, genes encoding enzymes and transcription factors (TFs) responsible for the biosynthesis, and the regulation of orchid floral volatiles. We hope that our information will provide guidance for future studies on orchid floral scents.

## 2. Orchid Volatile Compounds and Biosynthetic Pathways

**Figure 1:**
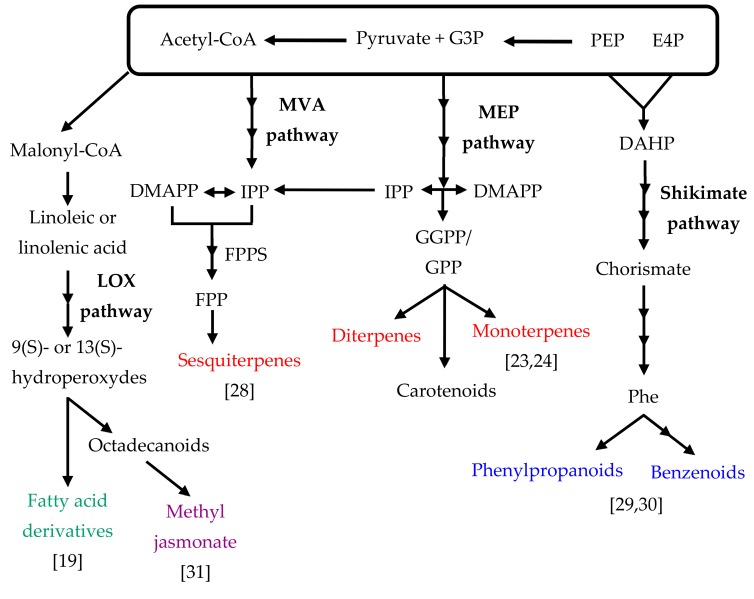
Floral volatile compound responsible pathways in orchid flowers. Major orchid floral volatile compounds are highlighted in colors (sesquiterpenes [28], monoterpenes [23,24], phenylpropanoids/benzenoids [29,30] and fatty acid derivatives/methyl jasmonate [19,31]). Abbreviations: MVA: mevalonic acid; MEP: methyl erythritol phosphate; LOX: lipoxygenase; PEP: phosphoenolpyruvate; G3P: glyceraldehyde-3-phosphate; E4P: erythrose 4-phosphate; DMAPP: dimethylallyl pyrophosphate; FPPS: farnesyl pyrophosphate synthase; FPP: farnesyl pyrophosphate; GGPP, geranylgeranyl pyrophosphate; GPP, geranyl pyrophosphate; IPP: isopentenyl pyrophosphate; DAHP: 3-deoxy-D-arabinoheptulosonate-7phosphate; Phe: phenylalanine.

Plant volatile compounds are a complex mixture of low molecular weight lipophilic molecules that have low melting points [27]. Biosynthesis of VOCs depends on the availability of carbon, nitrogen, and sulfur together with energy provided from the primary metabolism. Flower color and volatile compounds are key characteristics in many floricultural crops. Depending on their origin and functions, floral volatile compounds are categorized into one of three groups: terpenoids, phenylpropanoids/benzenoids, and fatty acid derivatives [20] (Figure 1).

### 2.1. Terpenoids

Terpenoids, or terpenes, represent the largest group of plant floral volatiles [27]. They play key roles in attracting pollinators for successful reproduction [32,33] and in defense against pathogens and florivores [34,35]. Moreover, from their natural roles, terpenoids are widely used in the cosmetic and perfume industries and as food additives because of their distinctive aromas and flavors [36,37]. Studies on floral scents have mainly focused on isolation and characterization of *terpene synthase* (*TPS*) genes encoding the key enzymes responsible for the synthesis of terpenes. All terpenoids are produced from isopentenyl diphosphate (IPP) and dimethyl allyl diphosphate (DMAPP), which are five-carbon (C5) precursors [38]. IPP and DMAPP are derived from two alternative biosynthetic pathways localized in different cellular compartments. The classical mevalonic-acid (MVA) pathway, which is localized in the cytosol, gives rise to IPP from three molecules of acetyl-CoA. In contrast, the methylerythritol phosphate (MEP) pathway takes place in plastids and produces IPP from pyruvate and glyceraldehyde 3-phosphate. In plants, monoterpenes, diterpenes, carotenoids, ubiquinones, and phytols are produced in the plastid via the MEP pathway, while all other plant terpenoids (sesquiterpenes, triterpenes, and polyterpenes) are produced using the MVA pathway. Floral volatiles with TPSs have been identified in such orchids as *P. bellina* [24] and *C. goeringii* [28].

Terpenoids are dominant in floral volatiles including those emitted by orchids. Geraniol, linalool, and their derivatives are major compounds of scented *P. bellina* flowers. Monoterpenes (Table 1) play a key role in the volatile profile [23,24]; in *C. goeringii*, floral volatile organic compounds include farnesol, methyl epi-jasmonate, (*E*)-β-farnesene, and nerolidol. Sesquiterpenes play a key role in the scent profile [28]. In the *Cymbidium* hybrid “Sunny Bell,” linalool is the major compound found in the petal [39]. The volatile floral scents inside species and cultivars of *Cymbidium* have been reported [28,39]. Among the volatiles α-pinene, eucalyptol, trans-β-ocimene, α-copaene, and β-caryophyllene terpenoid were leading components in the volatile mixture [40,41]. The Vanda Mimi Palmer flower mainly contains cimene, linalool oxide, and linalool, which are classified as monoterpenes [42]. In addition, nerolidol is a sesquiterpene [40,41]. Compared with model plants, there are few reports on floral scent terpenoids in orchids.

### 2.2. Phenylproponoids and Benzenoids

Phenylpropanoids and benzenoids are the second most dominant group of volatile compounds [14]. Phenylpropanoids/benzenoids are produced from the aromatic amino acid phenylalanine, which is produced in plastids via the shikimate pathway and arogenate pathway (Figure 1) through seven and three enzymatic steps [20]. Phenylalanine ammonia lyase (PAL) catalyzes the conversion of phenylalanine to trans-cinnamic acid, as a step in the phenylpropanoid pathway of plants [45]. The conversion of cinnamic acid to phenylpropanoids/benzenoids is followed by a shortening of the propyl chain via either the β-oxidative pathway or the non-β-oxidative pathway [45]. Recently, it was reported that the β-oxidative pathway for the formation of benzoic acid (BA) and benzenoids contributes to the production of volatile benzenoids in petunia flowers [46,47,48,49]. Moreover, in the fatty acid metabolism starting from cinnamic acid activation to its CoA thioester, hydration, oxidation, and thiolysis occurs in the peroxisome.

The formation of benzenoids (C6-C1) from cinnamic acid requires a shortening of the propyl side chain by two carbons and has been shown to proceed via a β-oxidative pathway, a non-β-oxidative pathway, or a combination of these pathways. Benzaldehyde formation, through the non-β-oxidative pathway, is oxidized by NAD+-dependent benzaldehyde dehydrogenase to benzoic acid, which is isolated from snapdragon flowers [50]. However, the formation of benzaldehyde in the enzymatic steps of the non-β-oxidative pathway remains unknown. Furthermore, floral phenylpropanoid and benzenoid compounds play a crucial role in scent production via two super families, SABATH methyl transferases and BAHD acyltransferases, in several plants [20]. In contrast, the formation of floral volatile phenyl propenes such as eugenol and isoeugenol starts with lignin and takes two enzymatic steps; the oxygen functionality at the C9 position is removed, and coniferyl alcohol is produced [20]. Finally, eugenol and isoeugenol are formed through the conversion of coniferyl acetate. From the *Cymbidium* cultivar Sael Bit, benzenes were reported in the full blooming stage [29]. Floral volatiles such as phenylpropanoids and benzenoids are also emitted from scentless flowers of *P. equestris*. In addition, *Gymnadenia* species quantitatively and qualitatively release diverse blends of nearly 50 volatile compounds and attract different suits of pollinators. Eugenol and benzyl acetate are two predominant compounds among the scents of these species [51].

### 2.3. Fatty Acid Derivatives

Among the floral volatile compounds, fatty acid derivatives are the smallest group of volatiles. They mainly consist of floral fatty acids synthesized from C18 polyunsaturated fatty acids and linolenic and linoleic acids. Methyl jasmonate is an important volatile fatty acid compound found in orchids. The biosynthetic process of fatty acid or its derivatives begins from the stereo-specific oxygenation catalyzed by lipoxygenases (LOXs) to produce 9-hydroxy and 13-hydroperoxy intermediates. These intermediates can enter two different batches of the LOX pathway to produce volatile compounds. Allene oxide synthase (AOS) catalyzes the first step in the biosynthesis of jasmonic acid from lipoxygenase-derived hydroperoxides of free fatty acids. In addition, the AOS pathway generates the C6 and C9 aldehydes through condensation of hydroperoxide derivatives by hydroperoxide lyase (HPLS). Limited data are available regarding the synthesis/pathways of fatty acids and/or their derivatives in flowers. In *Antirrhinum majus* flowers, 20 fatty acid derivatives have been identified [52]. Furthermore, methyl jasmonate and jasmonic acid involved in the floral scent pathway in *C. ensifolium* and *C. faberi* have been identified. Various volatile fatty acids were also found synthesized in the orchid genus *Ophrys*; among them, *alkenes* have an important function *in* *attracting pollinators* [17]. Two genes encoding stearoyl-acyl carrier protein desaturase (SAD) isoforms, SAD1 and SAD2, were reported to be flower-specific, and these genes broadly parallel alkene production during flower development of *Ophrys sphegodes* and *O. exalanta*; in particular, SAD2 showed a tight association with alkene production [19]. Further study is required to better understand the floral scent pathways in orchids.

## 3. Transcriptional Factors in Floral Volatile Regulation

Transcription factors (TFs) are sequence-specific DNA-binding proteins that interact with the regulatory regions of the target genes and modulate the transcription initiation rate by RNA polymerases [53]. Although several types of transcription factors have been reported to be involved in the biosynthesis of volatile compounds and secondary metabolites in plants, a limited number of TFs involved in the formation of molecules responsible for floral scent have been identified. Recently, a growing number of research results have reported that several types of TFs, including basic helix-loop-helix (bHLH), basic leucine zipper (bZIP), ethylene response factor (ERF), NAC, MYB, and WRKY family members, are involved in regulation for terpene biosynthesis [53].

TFs play a key role in controlling the expression level of genes involved in various developmental processes and physiological pathways, including plant secondary metabolism [54]. *ODORANT1* (*ODO1*) is the first *MYB* TF identified in flowers. It is a member of the R2R3-MYB TF family, which regulates genes involved in floral scent production through the shikimate and phenylpropanoid pathways [43]. Terpene biosynthesis is regulated by genes encoding required enzymes and their regulators. *Phalaenopsis* TFs regulate the terpenoid pathway; *PbbHLH4* regulates the *geranyl diphosphate synthase* (*GDPS*) gene for the synthesis of monoterpenoids in *P. bellina* [55]. Moreover, a higher expression of five genes encoding TFs (*PbbHLH4*, *PbbHLH6*, *PbbZIP4*, *PbERF1*, and *PbNAC1*) was reported in the scented orchid. Especially, 10-fold higher levels of α-terpineol (a monoterpenoid) were detected in *PbbZIP4*-overexpressing flowers of *P*. *aphrodite* compared to the control [56]. *HY5*, an *bZIP* TF, is known to play a critical role in mastering both the light and circadian signaling pathway. Identification of HY5-interacting motifs on the upstream regulatory fragments of *PbNAC1* implies that the light and circadian clock signals are likely to manage monoterpene biosynthesis in *P. bellina* [56].

Transcriptomic analyses of *C*. *goeringii* flowers led to identification of 1179 genes that were clustered into 64 groups encoding putative TFs with the three largest being bHLH (73 members), ERF (71 members), and C2H2 zinc finger proteins (65 members). *CgbHLH1* and *CgbZIP3* are homologs of *AabHLH1* and *AabZIP1*, respectively. *CgbZIP7* is a homolog of *PbbZIP4*, which regulates monoterpene biosynthesis in *P*. *bellina* (Table 2) [57], while *CgERF2* is a homolog of *CitAP2.10*, which is associated with sesquiterpene (+)-valencene synthesis in sweet orange [34]. *CgNAC5*, a homolog of *AaNAC4*, controls monoterpene synthesis in kiwifruit [35], and *CgWRKY1* and CgWRKY2, which are homologs of GaWRKY1, regulate sesquiterpene (+)-δ-cadinene synthesis in cotton [36]. Cymbidium Sael Bit MYB1 expression is detected at various flower developmental stages and is highest in petals and columns of the fully open flower. *Cymbidium* Sael Bit *MYB1* is regarded as a regulator of phenylpropanoid/benzenoid genes in floral scent profiles. The key component of flower scent in *C. faberi* is MeJA, which is regulated by the crosstalk of many plant hormones. A total of 379 TFs identified as belonging to 37 TF families are differentially expressed between blooming and withered flowers of *C. faberi* [58], and the top 10 groups belong to the *MYB*, *AP2-EREBP*, *bHLH*, *NAC*, *GRAS*, *C2H2*, *C2C2-Dof*, *MADS*, *WRKY*, and *ABI3VP1* families. Furthermore, an increase in the levels of floral volatiles in tissues resulted from a large increase of various transcription factors in orchids.

## 4. Spatial and Temporal Emission of Volatile Organic Compounds

Scent is an important property of flowers and plays a vital role in the ecological, economic, and aesthetic properties of flowering plants. Each plant possesses a distinct and unique floral scent. Floral scent is composed of all the VOCs, including terpenoids, phenylpropanoids, benzenoids, fatty-acids, and their derivatives, which are emitted by floral tissues (Table 2). In *Phalaenopsis bellina*, expression analysis of the *PbGDS* (*geranyl diphosphate synthase*) gene encoding a homodimeric GDS showed that its expression is flower-specific and that maximal expression is concomitant with maximal emission of monoterpenes on Day 5 post-anthesis [23]. In *P*. *abies*, expression of *PaIDS1* (*isoprenyl diphosphate synthase 1*) encoding a bifunctional GDS and GERANYLGERANYL DIPHOSPHATE SYNTHASE (GGDS) exhibits a peak in wood where oleoresin, comprising monoterpenes and diterpenes, is accumulated [24].

Plants emit a large variety of VOCs that are actively involved in plant growth and protection. VOCs are defined as any organic compound with vapor pressures high enough under normal conditions to be vaporized into the atmosphere [53]. VOC emissions are strongly dependent on environmental conditions and developmental stages of the plant tissue. In plants, emission of VOCs is spatiotemporally regulated; a majority of VOCs are emitted from flowers compared to other plant tissues/organs, and the level of emission increases when the floral bud is close to opening and decreases as it moves to the senescence stage [61,62].

In *Vanda* Mimi Palmer orchids, various types of sesquiterpenes and benzenoids were highly expressed at the full blooming stage with the expression of floral scent genes [42,59,63]. Floral volatile emission increased bud to flowering stages in *Cymbidium goeringii*. Different types of *Maxillaria* orchids emit strong vanilla or coffee-like scents, which are responsible for pollinator attraction [64]. *M. tenuifolia* Lindl is called a “coconut orchid” due to its strong coconut-like scent, and was recognized as the best scented orchid in the 18th World Orchid Conference [65]. The sepal is a source of floral volatiles in *Maxillaria* species, and the highest level of floral volatiles separated through electronic nose and GC-MS analyses was detected at the initial flowering stage [43,66]. In addition, methyl jasmonate (mJA) emission predominantly occurs in sepals and floral parts of *C*. *ensifolium* [31]. *Phalaenopsis* is undoubtedly the most widely grown orchid in the world, and in *P. bellina*, various types of monoterpenes are produced in the full flowering stage [23,24]. Furthermore, in a comparison of fragrant and non-fragrant *Phalaenopsis* flowers, terpene compounds were found to be much more abundant with increased levels of relevant gene expression in flowers of fragrant orchids [67].

Developmental regulation of scent emission occurs at several levels, including orchestrated expression of scent biosynthetic genes [68], enzyme activities, and substrate availability [69]. Based on an evolutionary study of floral scent genes in three closely related orchid species of the genus *Gymnadenia*, it is likely that the switch from the production of one to two scent compounds evolved under relaxed purifying selection [51]. Two major volatile compounds, α-copaene and β-caryophyllene, have been identified in all floral organs of *M. tenuifolia*, with the highest levels in the petal. α-copaene and β-caryophyllene were found to be emitted in all flower developmental stages except the floral bud stage I [43]. In fact, volatile compounds of *M. tenuifolia* include α-copaene, β-caryophyllene, 1,8-cineole, limonene, β-myrcene, α-pinene, β-pinene, sabinene, and δ-decalactone, which is responsible for the typical coconut aroma. The majority of studies on *Maxillaria* fragrance reported only the chemical composition of the floral scent; however, little data are available on the spatiotemporal emission of the floral volatiles. In addition, sulfur- and nitrogen-containing volatile compounds contribute to the attraction of pollinators to flowers by mimicking food or brood sources such as carrion or dung. Besides the importance of floral scents in plant ecology, identification and functional validation of relevant genes responsible for biosynthetic and/or regulatory pathways of floral volatiles are required for a better understanding of floral scent production and for the development of novel cultivars with desirable characteristics. Transcriptomic and metabolic analyses together with genetic engineering approaches will be of great help in driving towards this goal.

## 5. Gene Evolution for VOCs

The evolution of orchids has resulted in an immense diversity of flower traits such as color and scent. Orchidaceae consist of extraordinary adaptations that may have guaranteed its evolutionary success. To date, most of the examined plant gene families originated through gene duplication. Gene duplication plays a key role in species evolution because it provides raw materials for the evolution of new genes and new genetic functions. Multiple mechanisms contribute to gene duplication, including tandem duplication, segmental duplication, transposon-mediated duplication, and retro duplication. Studies of floral scent gene duplications in orchids have been limited. Orchid TPSs are the key enzymes that generate the structure diversity of terpenes. Through the analysis of plant genome, researchers have shown that the plant TPS gene family have their gene numbers ranging from 20 to 150 and thus belong to a mid-size family [70]. *Phalaenopsis equestris* genome has 23 TPSs belonging to TPS-a, -b, -c, e/f, and -g. Twenty-three TPSs found and predicted as having mono-, di- and sesqui-terpene synthase evolutionary relationships among orchids and experiencing duplication and then sub- or neo-functionalization, have occurred during evolution [71]. It has been proposed [72] that diterpene synthases are the origin of mono- and sesqui-terpene synthases during evolution. *P. aphrodite*, *PaCHS3*, *PaCHS4*, and *PaCHS5* formed a tandemly arrayed gene cluster, and the intervals between the three *CHS* genes were approximately 13.3 kb (*PaCHS3* and *PaCHS4*) and 7.7 kb (*PaCHS4 and PaCHS5*). This arrangement was also observed in a closely related orchid, *P. equestris*, in which the three CHS genes were all positioned on Scaffold 000036. Thus, the tandem array of three *CHS* genes was probably present in a common ancestor before speciation within *Phalaenopsis*. Tandem gene duplications represent a substantial proportion of all plant genes [73].

## 6. Functions of Orchid Volatile Compounds

**Figure 2:**
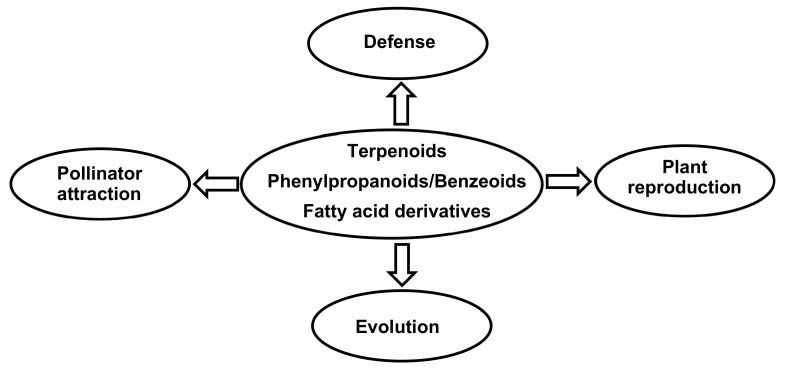
Functions of floral volatiles in orchid flowers.

Previous reviews provide a good summary of floral emissions and the involvement of biochemical processes in the interactions of flowers with their flower visitors [20], their action over pollinator behavior [21], and the ecological processes that drive their evolution [22], wherein they mediate intra- and interspecific interactions. The role of vegetative VOCs has been extensively reviewed [20]. Here, we present the functions of the floral VOCs, which are especially involved in the attraction of pollinators [20]. Floral volatiles have a role in many multifaceted functions that contribute to pollinator attraction, plant defense, plant reproduction, and plant diversity (Figure 2).

### 6.1. Flower Defense

Generally, flowers have effective physical barriers comprising highly lignified cell walls, although the generation of these cell walls renders flowers highly vulnerable to pathogens and florivores. Plants constitutively emit VOCs from flowers, leaves, and roots. Emission usually increases when plants are attacked by antagonists such as insect herbivores or pathogens [51].

Many VOCs were shown to exhibit antimicrobial and antifungal activities in vitro [74] or inferred to have these antimicrobial activities based on tissue-specific expression patterns [75]. However, only a few VOCs have been explored for their role in defense against pathogens. (*E*)-β-caryophyllene, emitted from stigmas of *Arabidopsis* flowers, was shown to limit bacterial growth; furthermore, *Arabidopsis* plants lacking (*E*)-β-caryophyllene emission displayed denser bacterial populations on their stigmas and reduced seed weight than wild-type plants, indicating that (*E*)-β-caryophyllene acts in the defense against pathogenic bacteria and is also important for plant fitness [76]. VOCs emitted by petals of *Saponaria officinalis* were also shown to inhibit bacterial growth, supporting their roles in controlling bacterial community diversity in petals [77].

### 6.2. Pollinator Attraction

In many flowering plant species, the emission of volatile scents from the flower is important for attracting insect pollinators. Orchid flowers exhibit visual, chemical, and morphological advertisements to guide their pollinators, and may offer rewards such as nectar, pollen, fragrance, or oil [78]. Over the past few years, evidence has supported the role of floral volatiles in pollinator attraction. In fact, a high occurrence of non-rewarding flowers has been noted in orchids compared to other plant families [79]. Floral volatile profiles are specific to each species depending on the type of pollinator [80]. However, the selection of pollinator has played a key role affecting the pattern of floral VOC profile across angiosperms.

Approximately one-third of all orchid species reach pollination over food deception, whereby flowers contain no nectar or other rewards but resemble or mimic floral signals of rewarding plants to attract pollinators [78]. Subsequently, intraspecific variation in floral traits is estimated to be high in food-deceptive orchids, since flowers must delay the avoidance learning of pollinators [79]. Flowers of the fly-pollinated *Satyrium pumilum* orchids emit a cocktail of six compounds containing sulfurous oligo sulfides such as dimethyl disulfide (DMDS) and dimethyl trisulfide (DMTS). Secretion of these volatiles is also tissue-specific, which is anticipated to be the key olfactory cue for attracting flesh-eating fly pollinators [80] and lepidopteran pollinators [81]. Floral scent can be distinguished even among closely related taxa when species differ in pollination systems, such as lepidopteran vs. bee fly pollination in *Narcissus* species [80] and bee vs. hummingbird pollination in two *Mimulus* species [80], suggesting that differences in the dominating functional group of pollinators drive divergence in floral scent.

### 6.3. Plant Reproduction

Pollinator attraction is often intermediated by multimodal signaling mechanisms including floral morphology, color, and scent [81]. In deceptive species, attractiveness is very important for ensuring reproductive success. For instance, *Ophrys* and *Neotinea* species are known to produce complex bouquets of volatiles typically consisting of more than 100 chemical compounds [82]. The species belonging to these genera are all deceptive, but *Ophrys* species use a sexual deception strategy, while *Neotinea* is a food-deceptive genus [83]. Various orchid volatiles play a key role in plant reproduction. In the Dracula orchid, *Dracula lafleurii*, the labellum acts as both a visual and an olfactory mimic of mushrooms that often grow alongside these orchids [84]. The labellum emits an unusual floral volatile blend of mushroom alcohols, especially (R)-1-octen-3-ol [85]. *Cypripedium calceolus* is pollinated by bees. Scent profile consists mainly of aliphatics, terpenoids, and aromatics. In this context, orchids are highly pertinent models for studying plant reproduction, as they present a great variety of floral traits and trait associations. This is mirrored by the great diversity of reproductive strategies in orchids. One of the most intriguing strategies is deceptive pollination (i.e., nectarless flowers), which is found in about one-third of orchid species. Floral scent analyses in *Ophrys orchids* [81] showed that their flowers emit attractive blends of VOCs. Moreover, different *Ophrys* species, which mainly use alkenes with certain double-bond positions as key signals for plant reproduction (e.g., non-hydrocarbons with low molecular weight), were found as “long-range” attractants [85].

Though by no means exclusive to orchids, deceptive pollination approaches are particularly well-developed in the Orchidaceae, with an estimated one-third of the family (around 10,000 species) using such strategies [85].

### 6.4. Evolution

Evolution of angiosperms has resulted in an immense diversity of flower traits such as shape, size, color, and scent. Remarkably, the quality and quantity of emitted volatiles are species-specific and vary among different populations within a species [20]. While much effort has so far been invested in describing scent composition in various flowering species, the mechanisms driving the evolution and diversification of floral scent remain underexplored. Analysis of the genetic basis for differences in scent profiles between these two species revealed that only two quantitative trait loci are responsible for the distinct scent phenotypes [86]. One of these locus maps to the MYB TF ODO1, which controls flux over the shikimate pathway and, therefore, the amount of precursors available for benzenoid biosynthesis [87], while the genetic identity of the second locus is presently unknown. *Ophrys* may rely on species-specific alkene emission profiles that are distinct in enzyme activity and on the gene expression of a few stearoyl acyl carrier protein desaturases of the *Ophrys* genus, and only limited genetic variation among species and populations was observed with microsatellite markers. These findings suggest that divergent pollinator-mediated selection rather than genetic drift explains the strong differences in volatile profiles. Taken together, the above examples demonstrate that small genetic variations can have large effects on floral scent chemistry and interactions with pollinators.

## 7. Case Studies of *Cymbidium* Floral Volatiles

Orchids are the largest, most highly diverse flowering plants, and form an extremely peculiar group of plants. *Cymbidium* is one of the most important genera of orchids for the cut-flower and potted plant markets. *Cymbidium* spp. have great horticultural value as ornamental plants because of their beautiful and fragrant flowers. The *Cymbidium* genus consists of nearly 55 species that are distributed mainly in tropical and subtropical Asia, reaching as far south as Papua New Guinea and Australia [88]. The *Cymbidium* genus can be divided into three subgenera (*Cymbidium*, *Cyperorchis*, and *Jensoa*) [89,90] and includes *C. sinense*, *C. goeringii*, *C. forrestii*, *C. faberi*, *C. ensifolium*, and *C. kanran*. *C. sinense* is a winter blooming epiphytic orchid usually regarded as a “Spring Festival” flower.

Great efforts have been made to better understand the flowering of orchids such as *Cymbidium*, *Phalaenopsis*, *Dendrobium* and *Cattleya* through biotechnological approaches including tissue culture and transgenic technologies [91,92,93,94]. Moreover, while *Cymbidium* orchid species are not all widely cultivated, hybrids of *Cymbidium* orchids lend themselves to cultivation. Some commercially important hybrids have been created for over 100 years. Because of their ornamental and commercial value, *Cymbidium* orchids have been the subject of taxonomic studies and, particularly, species identification [95,96,97]. In the past few decades, the application of diverse molecular techniques have contributed to widening our knowledge in the flowering/flower development, species identification, and volatile compounds of orchids.

### 7.1. Floral Volatile Research on Cymbidium

**Figure 3:**
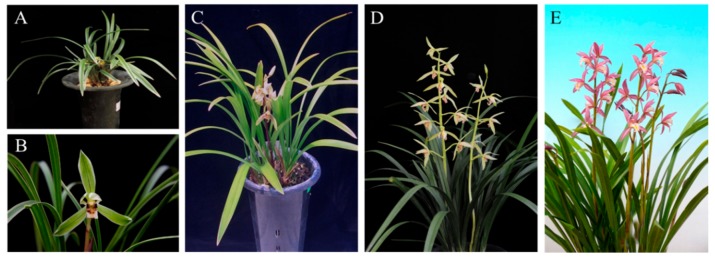
*Cymbidium* flowers described to the floral scent. (**A**) *C. goeringii*, (**B**) *C. faberi*, (**C**) *C. ensiforium*, (**D**) *C*. “Sael Bit,” (**E**) *C*. “Sunny Bell.”.

Floral VOCs are important compounds derived from flowers. Floral scent is a key trait for many floricultural crops. The molecular mechanisms underlying the regulation of biosynthesis and emission of volatiles from orchids remain largely elusive. For adding commercial value, studies on floral scents in orchids aim not only to understand the molecular and genetic mechanisms of the biosynthesis and emission of floral scents but also to assist in *Cymbidium* breeding programs. Currently, many orchid researchers are focusing on the development of cultivars with desirable floral scents. Recent reports on the propagation of sterile seedlings [98,99,100], leaf and flower morphogenesis [101,102,103], and the characterization of volatiles as floral scents [104,105] in *Cymbidium* spp. also reflect this trend. In fact, the biosynthesis of widespread VOCs in plant tissues is involved in multiple biological functions such as defense against pathogens, parasites, and herbivores [106,107,108]. The development of floral scents is likely to be a vital event in biological evolution, providing olfactory signals that plants can utilize to attract pollinators. We are presented major floral scent *Cymbidiums* (Figure 3).

#### 7.1.1. Cymbidium goeringii

*C. goeringii* is one of the most popular terrestrial species indigenous to temperate Eastern Asia, cultivated as an ornamental, and whose flowers are used as an ingredient in soup, alcoholic drinks, and tea [88]. Floral fragrance is determined by a mixture of volatile compounds. In *C. goeringii*, floral scent pathways have been studied in various developmental stages during flowering [28]. Sesquiterpenes are the major compounds in the *C. goeringii* floral scent profile. The dominant floral scent compounds were identified as farnesol, methyl epi-jasmonate, (*E*)-β-farrnesene, and nerolidol. In particular, examination of farnesol emission from the day of anthesis (D0) to the fifth day after anthesis (D+5) demonstrated that emission had a peak at the D+2 stage. Transcriptomic analyses focusing on floral scent pathways have been performed using three different stages of flowers in *C. goeringii*. Most terpenoid pathway genes, including *1-deoxy-D-xylulose-5-phosphate reductoisomerase* (*DXR*), *1-deoxy-D-xylulose-5-phosphate synthase* (*DXS*), and *farnesyl diphosphate synthase* (*FDPS*), were expressed at the initial flowering stage compared to bud and/or full flowering stages [28]. Furthermore, 32 and 38 unigenes known to be associated with MVA and MEP pathways, respectively, were reported in *C. goeringii.* Several TPSs and TFs were also identified in the floral transcriptome of *C. goeringii* including *CgTPS7*, which encodes a key enzyme involved in sesquiterpene synthesis. A putative terpenoid pathway responsible for the volatile profile in *C. goeringii* has also been reported.

#### 7.1.2. Cymbidium faberi

*C. faberi* Rolfe is one of the most significant species with elegant flower scents [44], and is one of the oriental orchids that has been longest cultivated. There are more than 100 compounds in the flower scent of blooming *C. faberi* flowers. Among these, MeJA is the most abundant, but is almost untraceable in the volatile emission of withered flowers [44]. The major pathways include α-linolenic acid metabolism, pyruvate metabolism, and fatty acid degradation, which contribute to the conversion of α-linolenic acid to MeJA. One of the differentially expressed genes (DEGs), *jasmonic acid carboxyl methyltransferase* (*CfJMT*), was highly regulated in the blooming flower of *C. faberi.* Consequently, the full-length coding sequence and genomic sequence of *CfAOS* from *C. faberi*, which is localized to the chloroplasts, has no introns, and is one of the most important enzymes in the MeJA biosynthetic pathways in *C. faberi* [58], was cloned. *CfAOS* has numerous roles including insect attraction and mediation of anti-microbial and stress tolerance. AOS and allene oxide cyclase (AOC) are crucial enzymes in the MeJA biosynthetic pathway in *C. faberi.*

#### 7.1.3. Cymbidium ensifolium

*C. ensifolium* is a popular miniature terrestrial orchid that produces fragrant flowers and is often marketed as a potted specimen. MeJA was one of the key scent compounds found in *C. ensifolium* [31]. It is known that MeJA is synthesized via the octadecanoid pathway. MeJA is primarily recognized as a floral scent compound in flowers of *Jasminum grandiflorum*, and is also universally distributed among the plant kingdom including many *Cymbidium* orchids [31]. MeJA emission was at very low levels in unopened or half-opened *C*. *ensifolium* flowers, and scent emission reached maximal levels between Days 4 and 6 and decreased from Days 7 to 10 post-anthesis. *C. ensifolium* tissue-specific manner and high MeJA emission was found in sepals and petals.

#### 7.1.4. *Cymbidium* Cultivar Sael Bit

The small green and light yellow colored flowers of *Cymbidium* cultivar Sael Bit are highly fragrant [109]. The strong emission of volatiles was detected in petals at the blooming stage. The dominant floral VOCs in Sael Bit were identified to be alkenes, benzenes, and estaric compounds; i.e., 2-methyl butanal, 2-methyl pentanal and (Z)-2-octenal at the blooming stage.

Full-length cDNA of *Cymbidium* Sael Bit *MYB1* from the petal has been isolated; fragrance genes such as *MYB1*, *OOMT*, *AOS*, *LOX*, and *SAMS* were expressed during the entire floral developmental stages, and all genes were highly expressed in the full opening flowers [29]. Sael Bit *MYB1* regulates the floral scent phenylproponoid- and benzenoid-responsible genes during scent emission.

#### 7.1.5. *Cymbidium* Cultivar Sunny Bell

A *Cymbidium* variety Sunny Bell (*C. karan* x *C. eburneum*) was developed at the National Institute of Horticultural & Herbal Science, Rural Development Administration, Suwon, Korea in 2013 [39]. Monoterpenes, sesquiterpenes, and aliphatics have been recognized as the major volatile compounds in *Cymbidium* Sunny Bell [39]. Twenty-four volatile components were identified in the Sunny Bell flowers; among the total volatiles, petals produced dominant volatile compounds compared to other floral tissues such as sepal, labellum, and column. Linalool is the major compound responsible for the floral volatile profile in Sunny Bell.

Some species in the genus *Cymbidium*, including *C. floribundum*, *C. pumilum*, and *C. suavissimum*, release identical volatiles for pollinator attraction. Various types of alkenes, esters, and fatty acid derivative compounds are released for pollinator attraction. It has been reported that *Cymbidium* flowers are rich in volatile compounds including cineole, isoeugenol, and (-) selinene [104]. Floral scent and color are major traits for floriculture crops in developing new cultivars of *Cymbidium*. Furthermore, 21–28 floral scent compounds have been identified as major volatile components in the flowers of three *Cymbidium* varieties [105]. The volatiles mainly comprise monoterpenes, aliphatics, and sesquiterpenes, and their content values have exceeded 90% [105]. Their aromatic characteristics can be determined by the profiles of each VOC that may vary depending on each genotype [105].

## 8. Final Remarks and Future Directions for Overcome the Challenges

**Figure 4:**
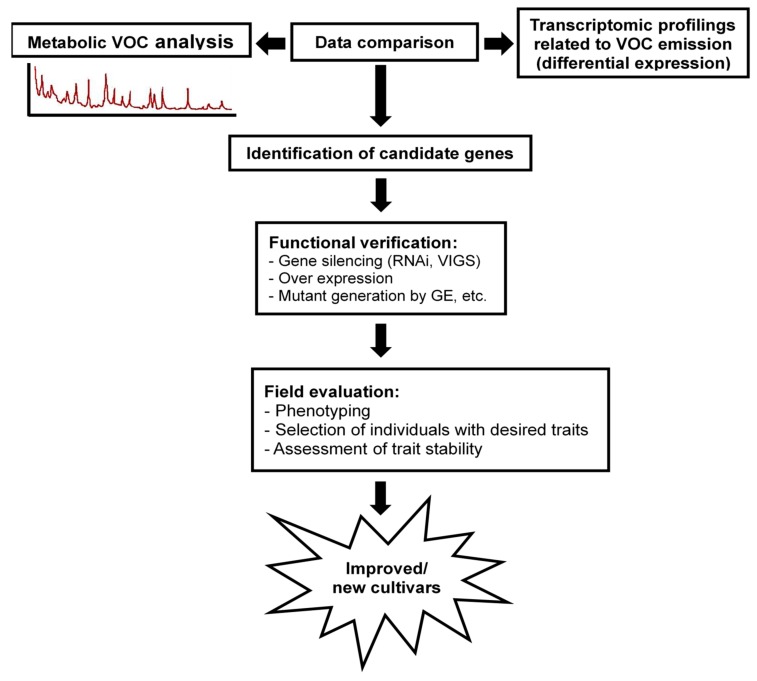
Schematic representation of functional studies for orchid breeding to develop floral scent trait.

At present, orchid industries worldwide are facing various difficulties. For developing new cultivars, physiological and genomic maps have been needed to produce markers. RNA-illumina sequencing technology has been extensively used for identifying gene expression at a genome-wide scale in many organisms, including non-model plants. The adoption of this technique, especially mRNA sequencing from floral tissues and de novo transcriptome construction (Figure 4), has been performed in several orchid species, with a goal of identifying genes involved in the biosynthesis and/or biosynthetic pathways of floral volatiles [28,110].

Moreover, other strategies include molecular evolutionary analysis tools. For example, testing for gene duplication and selection signatures in hypothesized pathway genes, from a phylogenetic perspective, is frequently used. For the identification of significant candidate genes and pathways, targeted and strategic transcriptome analyses of fragrant and non-fragrant flower organs and tissues are often the first key step. Regulation of DEGs between fragrant and non-fragrant tissues and developmental stages can be investigated. The latter provides a key baseline for identifying DEGs in fragrant tissues. This approach was utilized for the breakthrough discovery on volatile biosynthesis [58,111] and may hold potential for elucidating other biosynthetic pathways. RNA-sequencing analysis across species, with the goal of identifying shared gene expression and metabolic pathways, may also prove informative. Transformation technology has been developed for orchids; a few successful methods using virus induced gene-silencing (VIGS) approaches have recently been demonstrated as efficient strategies for functional studies of genes in orchids (Figure 4). Furthermore, transgenic approaches, such as the overexpression of floral scent genes and/or genome editing, have also been recently developed for orchids.

## 9. Conclusions

Over the last few decades, studies on plant volatile compounds and their biosynthetic processes have markedly increased. In orchids, volatile compounds play a key role in pollination, which ensures fertilization. To date, the biosynthesis of orchid floral fragrance is not well understood, with only some terpenoid pathways reported. Plant volatiles are generally produced at very low concentrations with low quantity, even in floral tissues. Thus, isolation of each component in the volatile compounds is inefficient and expensive. Despite the presence of studies of floral VOCs, many aspects of their biosynthesis together with transcriptional regulation and function require further studies. Further advances in functional studies on key genes for floral scent may rely on a breakthrough in orchid transformation technology that may lead to more efficient results. The genome sequences of several orchids have now been determined [112,113]. Overall, it is clear that genetic manipulation of orchid volatile compounds may be possible, but requires the selection of the appropriate species. In the future, studies in scent research may focus on orchid floral traits and on increasing phytochemical compounds, flavor, and aroma through the regulation of genes by transcription factors in floriculture crops. This review provides an important theoretical reference for aromatic volatile compound studies in orchids.

## Figures and Tables

**Table 1:** Major volatile organic components in orchid flowers.

Compound	Structure	Species	Reference
**Terpenoids**			
Linalool	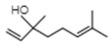	*P. bellina* *C*. cv. Sunny Bell	[23] [39]
Geraniol	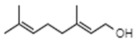	*P. bellina*	[24]
Ocimene	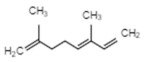	*Vanda* Mimi Palmer	[42]
Farnesol	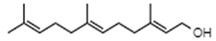	*C. goeringii*	[28]
β-Caryophyllene	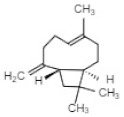	*M. tenufolia*	[43]
**Phenylproponoids/Benzenoids**			
Eugenol	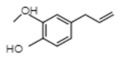	*Gymnadenia* Species	[30]
2-methyl butanal	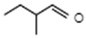	*C.* cv. Sael Bit	[29]
Benzyl acetate	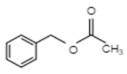	*Vanda* Mimi Palmer	[42]
**Fatty Acid Derivatives**			
Methyl jasmonate	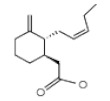	*C. ensifolium* *C. faberi*	[31] [44]

**Table 2:** Representative genes responsible for floral scents in orchids.

Floral Scent Gene	Metabolism Pathway	Species	Reference
**Genes**			
*PbGDS*	Terpenoid pathway	*Phalaenopsis bellina*	[24]
*VMPAAT*	Terpenoid pathway	*Vanda* species	[59]
*VMDXS*	Terpenoid pathway	*Vanda Mimi Palmer*	[42]
*GdEGS*	Benzenoid pathway	*Gymnadenia* species	[30]
*OsSAD1*	Benzenoid pathway	*Ophrys sphegodes*	[19]
**Transcription Factors (TFs)**			
*CsMYB1*	Phenylprponid/benzenoid	*Cymbidium* cv. Sael Bit	[29]
*PbbZIP4*	Monoterpene pathway	*Phalaenopsis aphrodite*	[60]
*PbBHLH2*	Monoterpene pathway	*Phalaenopsis bellina*	[23]
